# Double burden or double counting of child malnutrition? The methodological and theoretical implications of stuntingoverweight in low and middle income countries

**DOI:** 10.1136/jech-2017-209008

**Published:** 2017-05-31

**Authors:** Katie Bates, Arjan Gjonça, Tiziana Leone

**Affiliations:** Department of Social Policy, London School of Economics and Political Science, London, UK

**Keywords:** NUTRITION, MEASUREMENT, OBESITY

## Abstract

**Background:**

There is increasing concern at research and policy levels about the double burden of child malnutrition (DBCM)—with stunting and overweight found across different groups of children. Despite some case studies suggesting that stunting and overweight can occur concurrently in children, here known as ‘stuntingoverweight’, and major drives to reduce all forms of malnutrition in low and middle income countries (LMICs), stuntingoverweight is continually overlooked. This research evidences the prevalence of stuntingoverweight across LMICs, exploring the theoretical and methodological implications of failing to acknowledge this form of malnutrition.

**Methods:**

Prevalence estimates of stuntingoverweight are constructed from 79 LMICs with nationally representative anthropometric survey data. Stunting and overweight estimates are amended to exclude stuntedoverweight children. These estimates are compared with those published in the Joint Child Malnutrition Estimates (JMEs)—evidencing overestimation and double counting of stuntedoverweight children.

**Results:**

Children can be concurrently stunted and overweight. Stuntedoverweight children are found in all LMICs, from 0.3% to 11.7% of under-fives and are included in both stunting and overweight rates. Analysed together, this leads to double counting of stuntedoverweight children. This artificial inflation of stunting and overweight rates can give a false impression of a DBCM, obscuring the true diversity of malnutrition present. Over 10 million children are stuntedoverweight in the world.

**Conclusions:**

Stuntingoverweight is a newly recognised, understudied phenomenon. Affected children are included in both stunting and overweight prevalence estimates, introducing unobserved heterogeneity to both individual-level and population-level research and double counting to population-level research. Overlooking stuntedoverweight children has great implications for methodology, theory, policies, programmes and the health of affected children.

## Introduction

Child malnutrition remains a serious public health challenge. Globally, undernutrition rates have been falling, yet in 2015, 156 million children under-five were chronically undernourished.^1^ While levels of undernutrition remain unacceptably high, levels of overnutrition among under-fives have been increasing.^2^ In 2010, 38 million of the 43 million overweight under-fives were living in low and middle income countries (LMICs) and it is in these countries where the prevalence rates of overweight are increasing at the fastest rates.^3^ LMICs are said to be facing a ‘double burden of child malnutrition’ (DBCM). Children in these populations are at risk from either undernutrition or overnutrition; this has severe consequences for the affected individuals and for the long-term health of the country's population. However, these risks are not necessarily separate across individuals. In all LMICs, there are individual children affected by both undernutrition and overnutrition; these children are referred to in this paper as ‘stuntedoverweight’. This DBCM, at the individual-level, is an important public health problem and has notable implications for measurement, policy and interventions concerned with malnutrition.

Child undernutrition is a leading cause of ill health, disability and death; undernutrition is associated with 45% of all deaths among under-fives.^2^ Even in a child's early years, overnutrition is linked to elevated blood pressure and, later, increased risks of poor health in adulthood, notably non-communicable diseases (NCDs) including type II diabetes.^3^ ^4^ The faltering growth and development resulting from malnutrition cascade across the life course diminishing health and the social and economic opportunities of an individual.^5^

The eradication of hunger (severe and acute undernutrition) was an explicit millennium development goal target (MDG 1. Target 1c).^6^ While hunger has nearly halved between 1990 and 2015 in LMICs, these gains have not been matched for chronic undernutrition (stunting) and overnutrition, levels of which are currently higher than those of hunger.^7^ Indeed, overnutrition is thought to be increasing rapidly in almost all LMICs, while declines in stunting rates have stagnated in sub-Saharan Africa.^7^ In the post-2015 agenda, the sustainable development goals (SDGs) retain ending hunger and improving nutrition as an explicit aim (SDG2) and go further to highlight the role of improving nutrition for the realisation of all other SDGs.^8^

To monitor the DBCM and the increasingly complex burden of child malnutrition among populations today, multiple anthropometric indices (AIs) need to be used at once. Stunting and overweight (inter)national prevalence estimates are published routinely by UNICEF/WHO/World Bank Group in their Joint Child Malnutrition Estimates (JMEs) using two anthropometric indices—height-for-age (H/A) and weight-for-height (W/H). AIs are reported in terms of z-scores (SD points) from the median of a reference population, this reference population represents the growth of children in optimal environments.^9^ A child whose H/A is <−2SD below the median of this reference population has experienced severe growth faltering and is stunted as a result. A child whose W/H is >+2SD is overweight.^8^ As research moves to consider the population-level DBCM, these two AIs are presented to highlight the double burdens of undernutrition and overnutrition a population is experiencing.

However, a small number of studies have noted that stunting and overweight are not necessarily problems experienced by different individuals, across mother–child pairs at the household-level or among different population groups.^10–15^ Children can be concurrently stunted (H/A <−2SD) and overweight (W/H >+2SD), this nutritional profile is known here as stuntingoverweight. It is feasible that stuntingoverweight reflects a new layer of malnutrition that is resultant of rapid nutrition transitions (NTs) occurring in LMICs.^16^ Furthermore, each study shows stuntedoverweight children to be distinct from their stunted or overweight peers, not just by their nutritional profile. These initial studies suggest defining characteristics include living in rural, poor households and having overweight mothers.^10–15^

Beyond these initial studies, there is a lack of acknowledgement, comparable data and studies of stuntedoverweight children, across LMICs, and there are currently no national prevalence rates for stuntingoverweight available.^1^ This paper addresses the first of many gaps concerning stuntingoverweight by estimating the national prevalence of stuntingoverweight across LMICs.

The study objectives are to use nationally representative anthropometric data to (1) document stuntingoverweight among children under-five at the international level and (2) document the resultant ‘double counting’ of stuntedoverweight children at the population-level. Once documented, the paper moves to discuss (3) the implications of double counting for malnutrition research, (4) whether stuntingoverweight should be included in stunting and overweight rates, and (5) the theoretical implications of stuntingoverweight.

## Data

The latest data from three data sources are used—Demographic and Health Surveys (DHS), UNICEF Multiple Indicator Cluster Surveys (MICS) and the JMEs.

The most recent DHS or MICS surveys collecting anthropometric data for under-fives, between 2002 and 2017, are used by the authors to make their own ‘amended estimates’ (AEs) of stunting and overweight, excluding stuntingoverweight. These are compared with the JMEs, the largest freely available repository of child growth and malnutrition data in the public domain. Given its high impact value to the nutrition research community, it has been selected for use to compare with the AEs.

Surveys are only eligible for inclusion if the ‘final’ stunting and overweight prevalence rates (not ‘pending reanalysis’), sample size and age range are published in the latest edition of the JMEs.^17^ These surveys must also be publicly available for download from their original source—The DHS Program or UNICEF MICS, to enable the construction of the AEs. The most recent DHS or MICS surveys, by country, fulfilling this criteria are included in the study, yielding a sample of 79 LMICs (n=79).

## Methods

Prevalence rates of stuntingoverweight among under-fives are created for all countries from 25 MICS and 54 DHS surveys. Prevalence rates are constructed using z-score data in the MICS and DHS surveys that were created using the WHO 2006 Child Growth Standards.^9^ Children are classified as stuntedoverweight when they have an H/A of <−2SD and W/H of >+2SD.

Using the same z-scores, AEs of stunting and overweight are created that exclude stuntedoverweight children. Children are classified as stunted if they have an H/A of <−2SD and their W/H <+2SD and as overweight if they have a W/H >+2SD, with an H/A >−2SD. Data are weighted according to each survey’s protocols to provide nationally representative estimates of stuntingoverweight, stunting and overweight. In line with WHO recommendations, biologically implausible z-scores of <−5 or >+5 for WHZ and/or <−6 or >+6 for HAZ are flagged and affected cases excluded from the analysis.^9^ Prevalence estimates are created for the same age range of children under-five as published in the JMEs.

To document the hypothesised double counting of stuntedoverweight children, the methodology used by the WHO to create national estimates is reviewed for any recommendations to deal with overlapping AIs.

## Results

The AEs are presented in table 1,^17–20^ next to the current rates published in the JMEs prevalence of stunting and overweight for all countries included in the study. SE and 95% CIs of the AEs are available in online supplementary table S1.^18^ ^19^

10.1136/jech-2017-209008.supp1 supplementary data

**Table 1:** Percentages of children stunted, overweight or stuntedoverweight in 79 LMICs (JMEs and AEs)*

		JMEs (%)^17^		Amended estimates (AEs) (%)^18,19^		
Country	Year	Stunting†	Overweight	Stunting†	Overweight	Stuntingoverweight
Albania	2008	23.1	23.4	10.2	12.6	9.0
Armenia	2010	20.8	16.8	12.2	8.2	7.1
Azerbaijan	2006	26.8	13.9	16.6	4.3	8.6
Bangladesh	2011	41.4	1.9	40.5	0.9	0.7
Barbados	2012	7.7	12.2	6.1	9.6	1.6
Belarus	2005	4.5	9.7	3.4	8.8	1.0
Belize	2011	19.3	7.9	17.1	5.5	2.3
Benin	2006	44.7	11.4	36.1	2.3	6.9
Bhutan	2010	33.6	7.6	28.2	2.1	5.4
BiH	2011–12	8.9	17.4	5.1	13.0	3.8
Bolivia	2008	27.2	8.7	24.6	6.0	2.5
Burkina Faso	2010	35.1	2.8	33.1	1.0	1.4
Burundi	2010	57.5	2.9	56.0	0.8	1.9
Cambodia	2010	40.9	1.9	38.1	0.7	1.0
Cameroon	2011	32.6	6.5	29.5	3.8	2.4
Central African Republic	2006	45.1	8.5	37.6	3.3	5.0
Chad	2004	44.8	4.4	42.4	1.7	2.2
Colombia	2010	12.7	4.8	12.6	4.3	0.5
Comoros	2012	32.1	10.9	25.0	4.4	4.7
Congo (Brazzaville)	2011–12	25.0	3.6	21.7	1.9	1.4
Congo Democratic Republic	2013–14	42.6	4.4	39.9	1.6	2.5
Djibouti	2006	32.6	13.4	26.3	5.4	7.3
Dominican Republic	2013	7.1	7.6	5.9	6.5	1.0
Egypt	2014	22.3	15.7	13.9	7.3	7.6
Ethiopia	2011	44.2	1.8	43.5	0.9	0.9
Gabon	2012	17.5	7.7	13.7	5.1	2.3
Gambia	2005	27.6	2.7	26.5	1.6	1.1
Georgia	2005	14.7	21.0	6.9	14.0	6.6
Ghana	2008	28.6	5.9	25.0	2.8	2.4
Guinea	2012	31.3	3.8	29.3	2.0	1.6
Guinea-Bissau	2006	47.7	17.0	35.7	3.3	11.7
Guyana	2009	19.5	6.7	16.6	4.6	2.1
Haiti	2012	21.9	3.6	19.7	2.6	1.1
Honduras	2011–12	22.7	5.2	21.8	4.7	0.6
India	2005	47.9	1.9	47.1	0.6	1.0
Iraq	2006	27.5	15.0	19.0	7.2	7.4
Ivory Coast	2011	29.6	3.2	28.5	1.7	1.3
Jordan	2012	7.8	4.7	7.1	3.9	0.5
Kazakhstan	2010–11	13.1	13.3	8.5	8.5	4.7
Kenya	2008–09	35.2	5.0	32.9	2.3	2.4
Kyrgyzstan	2012	17.8	9.0	14.2	5.1	3.5
Lao PDR	2011–12	43.8	2.0	42.7	0.8	1.3
Lesotho	2009–10	39.0	7.3	34.5	4.5	3.1
Liberia	2013	32.1	3.2	28.9	1.5	1.4
Madagascar	2003–04	52.8	6.2	48.9	1.3	4.1
Malawi	2010	47.8	9.2	42.0	3.1	5.1
Maldives	2009	20.3	6.5	16.8	4.6	1.2
Mali	2006	38.5	4.7	35.7	2.0	2.1
Mauritania	2011	29.7	3.2	26.7	1.4	1.8
Mongolia	2010	15.6	4.7	13.1	8.8	2.0
Montenegro	2013	9.4	22.3	4.0	15.5	5.7
Morocco	2003–04	23.1	13.3	17.0	7.6	5.5
Mozambique	2011	43.1	7.9	38.2	2.8	4.6
Namibia	2013	23.1	4.1	21.1	2.6	0.9
Nepal	2011	40.5	1.5	39.6	0.8	0.7
Niger	2012	43.0	3.0	41.9	1.1	1.4
Nigeria	2013	36.4	4.9	34.1	1.3	2.6
Pakistan	2012–13	45.0	4.8	42.1	1.0	2.3
Peru	2012	18.4	7.2	17.4	6.9	0.5
Rwanda	2010	44.3	7.1	40.4	3.3	3.6
Sao Tome e Principe	2008	31.6	11.6	23.7	5.2	5.7
Senegal	2014	19.4	1.3	20.0	0.7	0.3
Serbia	2010	6.6	15.6	3.6	12.3	3.0
Sierra Leone	2013	37.9	8.9	33.5	3.4	4.3
Somalia	2006	42.1	4.7	39.7	2.1	2.2
Suriname	2010	8.8	4.0	13.1	8.8	2.0
Swaziland	2010	31.0	10.7	28.1	7.8	2.8
Syria	2006	28.6	18.7	16.9	7.1	10.9
Tajikistan	2012	26.8	6.6	23.0	2.7	3.1
Tanzania	2009–10	42.5	5.5	39.0	2.5	2.6
Thailand	2005–06	15.7	8.0	13.7	6.1	1.9
Timor-Leste	2009	57.7	5.8	54.1	1.1	3.6
Togo	2010	29.8	1.6	29.4	1.3	0.3
Turkey	2003–04	15.6	9.1	13.9	7.5	1.2
Uganda	2011	33.7	3.8	31.4	2.0	1.7
Uzbekistan	2006	19.6	12.8	14.7	8.0	4.2
Vanuatu	2007	25.9	4.7	23.2	1.6	2.4
Zambia	2013–14	40.0	6.2	36.6	2.5	3.3
Zimbabwe	2010	32.3	2.8	29.1	3.2	2.0

*It should be noted that any discrepancies between the Joint Child Malnutrition Estimates (JMEs) stunting and overweight rates and respective AEs (including stuntedoverweight children for consistent comparison) are thought to be, in the main, due to differential cleaning criteria in the JMEs compared with the AEs.^20^ For example, in the case of Kazakhstan, dropping biologically implausible cases for the analysis for AEs led to a sample size of 4985 children. In the JMEs, n=5015.^17^ Although this type of issue has been documented elsewhere,^20^ the online supplementary tables S2 and S3 17–21 provide a description of this issue and detail the differences in sample size and estimates.

†In line with the JMEs, AEs of stunting include children who are concurrently stunted and wasted, this has impact on estimates of stuntingoverweight or overweight.

Stuntedoverweight children are found in every single country in the analysis. Guinea-Bissau has the highest prevalence of stuntedoverweight children, 11.7%, the lowest prevalence is found in Senegal, 0.3%.

A review of the methodology of the JMEs database highlights that an individual's result on H/A are not considered when creating overweight prevalence rates (this is also true of stunting rates and W/H).^22^ This, in addition to the AEs in table 1, confirms that stuntedoverweight children are currently included in both stunting and overweight estimates. To illustrate this, the case of Kazakhstan can be considered. According to the JMEs, in 2010–2011, stunting was 13.1%, overweight 13.3% (table 1). Kazakhstan's AEs show 4.7% of children are stuntedoverweight, these were included in both stunting and overweight JMEs. Excluding the stuntedoverweight, stunting rates are now 8.5% (≈13.1 minus 4.7) and overweight 8.5% (≈13.3 minus 4.7%) (table 1).^17–21^ Thus, currently, when research involves using both stunting and overweight levels for a population, these data contain double-counted stuntedoverweight children.

**Figure 1:**
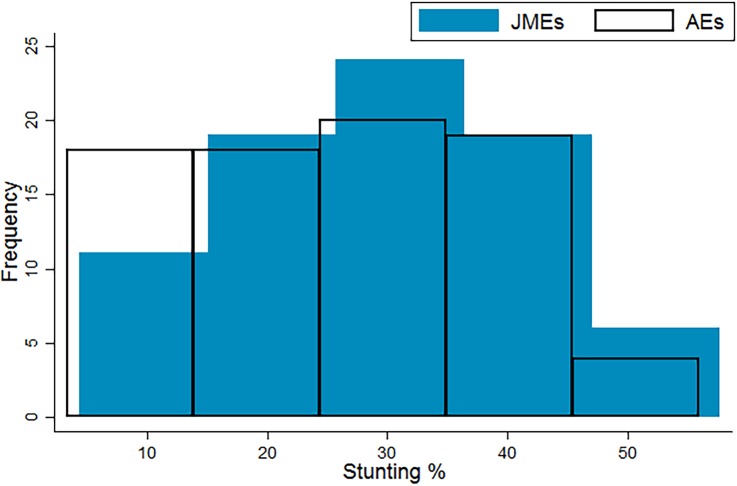
Current (JMEs) and amended estimates (authors) of stunting in 79 LMICs.^17–19^ JMEs, Joint Child Malnutrition Estimates; LMICs, low and middle income countries.

The AEs of stunting, excluding stuntingoverweight, range from 3.4% in Belarus to 56.0% in Burundi in the sample, compared with a range of 4.5% in Belarus to 57.7% in Timor-Leste using the JMEs. The JMEs of stunting show a normal distribution across the LMICs, while the AEs show a mild positive skew, relative to the JMEs (figure 1).^17^ ^18^ ^19^ Clearly excluding stuntingoverweight from the prevalence rates will reduce the magnitude of stunting prevalence, yet the change in distribution also shows that stuntingoverweight prevalence is not evenly distributed, relatively or absolutely, across LMICs. Within the sample, the inclusion of stuntedoverweight cases is increasing stunting prevalence rates by between 1.0% (Togo) to 142.5% (Montenegro). Ten countries in the sample see their stunting rates inflate by over 50% by including stuntedoverweight children.

**Figure 2:**
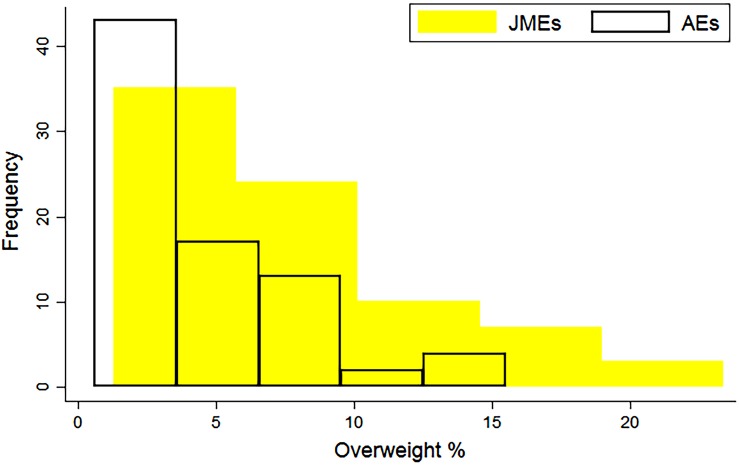
Current (JMEs) and amended estimates (authors) of overweight in 79 LMICs.^17–19^ JMEs, Joint Child Malnutrition Estimates; LMICs, low and middle income countries.

AEs of overweight range from 0.6% in India to 15.5% in Montenegro. The JMEs range is from 1.3% in Senegal to 23.4% in Albania (table 1). Both JMEs and AE rates of overweight are positively skewed, but the AE distribution is narrower, with a lower median (figure 2).^17–19^ There are relatively lower levels of overweight (compared with stunting) in most LMICs, yet a constant level of stuntingoverweight, thus the inclusion of stuntedoverweight children is leading to a greater increase in the prevalence of overweight. The increase is between 7.2% (Peru) to 354.5% (Guinea-Bissau). Thirty-six countries (45.5%) are seeing their overweight rates more than double by counting stuntedoverweight children as ‘overweight’ as in the JMEs.

The extent of the distance between stunting and overweight prevalence created by excluding stuntedoverweight varies across LMICs but the inflation stuntingoverweight creates for both prevalence estimates is apparent in all LMICs (table 1).^17–21^ In absolute numbers, using estimates of the population of children under-five for these countries' respective years, over 10 million children are found to be concurrently stunted and overweight.^17^ ^18^ ^19^

## Discussion

For the first time, the prevalence of stuntingoverweight across LMICs has been documented. This has revealed that the individual-level DBCM, stuntingoverweight, is not an idiosyncratic phenomenon found in a small number of populations, but is found among children under-five in all LMICs. Over 10 million children are estimated to be stuntedoverweight. In over 40% of the LMICs in this study, there are more stuntedoverweight children than overweight children. These findings have clear implications for our understanding of child malnutrition in LMICs today.

The JMEs for overweight and stunting both currently include stuntedoverweight children. Aggregate-level DBCM research using the JMEs double counts children. Double counting can introduce unobserved heterogeneity into analyses, and bias results, as shown in this paper. The emerging agenda focusing on tackling the DBCM is relying on data that is exaggerating and polarising burdens of child malnutrition in many countries and, further, marginalising stuntedoverweight children. When assessing child malnutrition at the population-level using AIs, researchers must consider all forms of malnutrition, including those where the indices overlap, notably stuntingoverweight and, further, children who are concurrently stunted and wasted.

Stuntedoverweight children are currently automatically included in both stunting and overweight rates. This leads to increases in each individual rate of up to 143% for stunting and 354% for overweight. When used individually, these rates do not double count stuntedoverweight children, but there is no current discussion on whether stuntingoverweight should be conflated with either stunting or overweight. Stuntedoverweight children are indeed ‘truly’ stunted, as they have not reached the expected velocity in their height for their age. They are, however, also overweight. Further research is needed to develop a consensus on how to present population-level stunting rates, given stuntingoverweight. Research is needed to justify the inclusion, or exclusion of stuntingoverweight in stunting rates at the population-level. If stuntedoverweight children should not be included in stunting prevalence, stunting is overestimated. Given studies already highlighting unique determinants of stuntedoverweight children,^10–15^ research on stunting at the individual-level should routinely include stuntingoverweight as an independent parameter.

For overweight, the inclusion of stuntingoverweight is highly questionable. These children are experiencing overnutrition but have also suffered chronic undernutrition; stuntedoverweight children are arguable not ‘truly’ overweight, they have a double nutritional insult. This is particularly concerning in the context of physiological studies indicating that it is stunting in early childhood and altered fat metabolism which is leading to increased central adiposity in later childhood and adolescence, among stunted children.^23^ Further research is needed to assess if stuntingoverweight among under-fives reflects an even earlier onset of the effects of stunting on central adiposity, and if, in clinical settings a stuntedoverweight child requires treatment distinct to that of overweight children, and indeed stunted children.

At the population-level, the inclusion of stuntedoverweight children in overweight rates is skewing our understanding of the current burden of overnutrition—this distortion is great (figure 2), a threefold overestimate in the case of Guinea-Bissau.^17–19^ This is particularly important in the context of a policy agenda concerned with the rapid increase in overnutrition in LMICs, research is needed to establish if this ‘rapid increase’ is an artefact of including stuntedoverweight children in overweight rates, or if both stuntingoverweight and overweight are increasing. In addition, given stuntingoverweight, the velocity at which ‘pure’ overweight is increasing needs to be reassessed.

The neglected status of stuntingoverweight in research means the determinants of this specific group of children are largely unexplored. Determining the pathways to stuntingoverweight is a key area for future research. Four case studies on stuntingoverweight suggest the developmental trajectory of stuntingoverweight is sequential; a child becomes stunted first and then overweight. These studies propose diet, low physical activity levels, rapid postnatal weight gain and socioeconomic status as factors that can lead to the development of stuntingoverweight in contexts of ongoing nutrition transitions.^10–15^ ^24^ A key question that remains unanswered is the relation to intergenerational effects and the Barker hypothesis; whether epigenetic alterations are affecting the metabolism, predisposing a stunted child to overweight.^25^ ^26^ The focus on the effects of these metabolic changes on increased risk of disease in adult life should widen to consider whether effects are apparent far earlier in the life course.^27^ If suboptimal in utero conditioning has created a predisposition for some stunted children to become overweight, that has become apparent during today's nutrition transition, when socioeconomic conditions improve, reducing the risk of disease and changing dietary patterns, the intergenerational effects of undernutrition are far more widespread than currently thought. Additionally, the consequences of stuntingoverweightness on health and development across the life course and thus on the health system and on economic productivity are currently unknown. Further research is needed in a wide range of countries and contexts to explore the developmental trajectory of, underlying mechanisms that lead to and consequences of stuntingoverweight. Understanding these will enrich our understanding of both the Barker hypothesis and the nutrition transition theory, and enable targeted interventions for children facing a dual nutritional insult to be developed.

Ultimately, for countries undergoing their NT today, a rapid, ‘altered trajectory’ means undernutrition remains within a population while overnutrition increases.^27^ ^28^ In these populations, many children are also experiencing an individual-level DBCM. Stuntingoverweight highlights that this ‘altered trajectory’ of NTs occurring in LMICs today is more complex than a simple polarised burden of overnutrition and undernutrition.^29^

## Conclusions

Driving the agenda to tackle child malnutrition, through nutrition programming and policies, is the Rome Declaration on Nutrition and the Framework for Action and UNICEF's Approach to Scaling Up Nutrition Programming for Mothers and their Children.^30^ Central to both strategic plans is accurate monitoring of malnutrition at the country level.^31^ ^32^ Currently, however, failing to acknowledge stuntingoverweight is leading to double counting of stuntedoverweight children and misleading representations of the true burdens of malnutrition faced by a population. To achieve more accurate monitoring of growth-faltering malnutrition:

- The overlap of AIs needs to be both acknowledged widely and quantified in published rates, particularly for stunting and overweight in the context of a DBCM.
- A consensus should be reached on how to present prevalence rates for overlapping forms of growth-faltering malnutrition, to avoid double counting.
- Anthropometric software should be adapted to routinely and consistently flag overlapping AIs.
- A greater level of transparency is required in the construction of AIs and prevalence rates; notably cleaning criteria, final sample size and details on how the data were actually weighted.

From the limited evidence available, initial indications are that stuntedoverweight children should be treated in nutrition research as a separate, distinct group—a result of a unique combination of maternal, socioeconomic and contextual factors.^10–15^ Stuntedoverweight children are a neglected area in public health, they are understudied and are outside of the scope of the most influential nutrition theories currently driving nutritional research and programmes. Further research into the determinants of stuntingoverweight is required. Understanding the development of concurrent paradoxical nutritional status provides an opportunity to further our understanding of the Barker hypotheses and nutrition transition theory, and importantly to address children who are truly suffering a double burden of malnutrition.^16^

Stuntedoverweight children are found in every LMIC in the study. With over 10 million stuntedoverweight under-fives in LMICs, research and policymaking should focus more on this distinctive group of malnourished children.

**What is already known on this subject**

- A small number of case studies have found children can be concurrently stunted and overweight, here known as stuntedoverweight; stuntedoverweight children have been reported in Russia, Mexico, Brazil, Cameroon, South Africa, Indonesia, Jamaica and China, as well as among specific ethnic groups (Hispanic-American and Andean populations). These studies suggest stunted children are at increased risk of overweight and obesity, with proposed risk factors for stuntingoverweight including malnourished mothers, poor diet and poor socioeconomic conditions. However, stuntingoverweight, its prevalence, and its effect on our understanding of child malnutrition has not been documented systematically across low and middle income countries (LMICs).

**What does this study add**

- This is the first study to document stuntingoverweight in LMICs, where data are available. Stuntedoverweight children are found in every LMIC in the study, from 0.3% of under-fives in Senegal to 11.7% in Guinea-Bissau. For the first time, this study shows that stuntedoverweight children are, currently, included in both stunting and overweight prevalence estimates. When researchers use stunting and overweight rates at an aggregate level, the data is double counting stuntedoverweight children, inflating both stunting and overweight rates. In over 45% of countries in the sample, there are more stuntedoverweight children than overweight children, challenging our understanding of the rise of ‘truly’ overweight children in LMICs. Stuntingoverweight has implications for the health and development of affected children, our current understanding of malnutrition in LMICs, current practices in defining and estimating growth faltering malnutrition and, subsequently, affects how malnutrition can be targeted and reduced.
