# Supplementary material for: Double burden or double counting of child malnutrition? The methodological and theoretical implications of stuntingoverweight in low and middle income countries
Source: J Epidemiol Community Health. 2017 May 31;71(8):779–85. doi: 10.1136/jech-2017-209008 (PMC5537509; doi:10.1136/jech-2017-209008)
Supplement: supplementary data [file jech-2017-209008supp001.pdf]

SUPPLEMENTARY MATERIAL

Supplementary Table 1: Standard Error (SE) and Upper Bound (UB) and Lower Bound (LB) of 95% Confidence Interval Estimates of AE Prevalence Rates[18,19]

Country	Year	Stunting			Overweight			Stuntingoverweight		
		SE	LB	UB	SE	LB	UB	SE	LB	UB
**Albania**	2008-09	1.1	8.3	12.6	1.2	10.4	15.2	1.0	7.2	11.3
**Armenia**	2010	1.2	10.0	14.8	1.0	6.5	10.3	0.9	5.5	9.0
**Azerbaijan**	2006	1.3	14.2	19.3	0.6	3.2	5.8	1.1	6.7	10.9
**Bangladesh**	2011	0.8	38.9	42.1	0.1	0.6	1.2	0.1	0.5	0.9
**Barbados**	2012	1.2	3.6	8.6	1.8	7.6	14.6	0.7	0.8	3.9
**Belarus**	2005	0.5	2.6	4.5	0.6	7.7	9.9	0.2	0.7	1.4
**Belize**	2011	1.4	14.3	19.8	0.6	4.5	6.8	0.5	1.4	3.3
**Benin**	2006	0.6	34.9	37.2	0.1	2.1	2.6	0.4	6.1	7.7
**Bhutan**	2010	1.0	26.5	30.3	0.2	1.7	2.7	0.4	3.8	5.5
**BiH**	2011-12	0.6	2.2	4.8	1.2	11.3	15.9	0.7	2.7	5.7
**Bolivia**	2008	0.9	22.8	26.4	0.4	5.3	6.8	0.2	2.1	3.0
**Burkina Faso**	2010	0.7	31.6	34.5	0.2	0.7	1.3	0.2	1.1	1.8
**Burundi**	2010	1.0	53.9	58.0	0.2	0.5	1.3	0.3	1.4	2.7
**Cambodia**	2010	1.1	35.9	40.4	0.2	0.4	1.1	0.2	0.6	1.5
**Cameroon**	2011	1.0	27.6	31.6	0.3	3.2	4.5	0.3	1.9	3.1
**Central African Republic**	2006	1.0	35.8	39.5	0.4	2.7	4.1	0.4	4.2	5.9
**Chad**	2004	1.1	40.2	44.6	0.2	1.3	2.1	0.4	1.6	3.0
**Colombia**	2010	0.4	11.8	13.4	0.2	3.9	4.7	0.1	0.4	0.7
**Congo (Brazzaville)**	2011-12	1.0	19.8	23.8	0.3	1.4	2.7	0.3	0.9	2.1
**Congo Democratic Republic**	2013-14	0.9	38.1	41.7	0.2	1.2	2.0	0.3	2.0	3.1
**Comoros**	2012	1.2	22.6	27.4	0.6	3.3	5.8	0.6	3.5	6.1
**Djibouti**	2006	1.5	23.4	29.4	0.8	4.0	7.1	0.9	5.6	9.3
**Dominican Republic**	2013	1.7	5.9	12.9	0.6	1.2	3.5	0.3	0.2	1.7
**Egypt**	2014	0.5	13.0	14.9	0.3	6.7	8.0	0.4	6.8	8.4
**Ethiopia**	2011	0.9	41.7	45.2	0.1	0.7	1.2	0.1	0.6	1.2
**Gabon**	2012	1.6	13.8	19.9	0.8	3.2	6.5	0.5	1.3	3.2
**Gambia**	2005	0.7	25.1	28.0	0.2	1.3	1.9	0.2	0.8	1.4
**Georgia**	2005	0.8	5.5	8.5	1.0	12.2	16.0	0.8	5.2	8.3
**Ghana**	2008	1.2	22.8	27.4	0.4	2.1	3.7	0.4	1.7	3.4
**Guinea**	2012	1.1	27.2	31.5	0.3	1.5	2.7	0.3	1.1	2.3
**Guinea-Bissau**	2006	1.0	33.9	37.7	0.3	2.8	4.0	0.6	10.6	12.8
**Guyana**	2009	1.6	13.8	19.9	0.8	3.2	6.5	0.5	1.3	3.2
**Haiti**	2012	1.0	17.8	21.8	0.3	2.0	3.3	0.2	0.8	1.6
**Honduras**	2011-12	0.7	20.5	23.1	0.3	4.1	5.3	0.1	0.4	0.8
**India**	2005	0.4	46.3	47.9	0.0	0.5	0.7	0.1	0.9	1.1
**Iraq**	2006	0.5	18.2	20.0	0.3	6.6	7.9	0.3	6.7	8.1
**Ivory Coast**	2011	1.1	26.4	30.6	0.3	1.2	2.3	0.2	0.9	1.9
**Jordan**	2012	0.6	6.0	8.4	0.4	3.1	4.8	0.1	0.4	0.8
**Kazakhstan**	2010-11	0.5	7.3	9.2	0.5	7.7	9.7	0.4	3.9	5.4
**Kenya**	2008-09	1.1	30.7	35.1	0.3	1.8	2.8	0.3	1.9	3.0
**Kyrgyzstan**	2012	0.8	12.7	15.9	0.4	4.3	6.0	0.4	2.8	4.5
**Lao PDR**	2011-12	0.9	41.3	44.7	0.1	0.6	1.0	0.1	0.8	1.3
**Lesotho**	2009-10	1.6	26.7	32.9	0.7	3.9	6.8	0.5	1.9	3.9
**Liberia**	2013	1.1	26.7	31.2	0.3	1.0	2.3	0.3	0.9	2.0
**Madagascar**	2003-04	1.6	45.8	52.0	0.2	0.9	1.9	0.6	3.1	5.4
**Malawi**	2010	1.0	40.1	43.9	0.3	2.5	3.8	0.4	4.3	5.9
**Maldives**	2009	1.0	14.9	18.8	0.5	3.7	5.7	0.3	0.8	1.8
**Mali**	2006	0.8	34.3	37.2	0.3	1.5	2.6	0.2	1.8	2.5
**Mauritania**	2011	0.7	26.8	29.7	0.2	1.2	1.9	0.2	1.2	1.9
**Mongolia**	2010	0.7	11.8	14.6	0.6	7.6	10.1	0.3	1.5	2.6
**Montenegro**	2013	0.4	0.8	2.6	1.5	13.7	19.5	1.4	3.5	9.1

**Morocco**	2003-04	0.7	15.6	18.5	0.4	6.9	8.5	0.4	4.8	6.2
**Mozambique**	2011	0.8	36.7	39.7	0.2	2.5	3.2	0.4	4.0	5.4
**Namibia**	2013	1.3	18.7	23.8	0.5	1.8	3.7	0.2	0.5	1.5
**Nepal**	2011	1.4	36.8	42.5	0.2	0.4	1.3	0.2	0.4	1.2
**Niger**	2012	1.0	39.9	43.9	0.2	0.8	1.5	0.2	1.0	1.8
**Nigeria**	2013	0.6	32.9	35.3	0.1	1.1	1.5	0.2	2.3	2.9
**Pakistan**	2012-13	1.4	39.3	44.9	0.2	0.6	1.5	0.3	1.7	3.1
**Peru**	2012	0.6	16.1	18.6	0.4	6.0	7.8	0.1	0.3	0.7
**Rwanda**	2010	0.9	38.7	42.2	0.3	2.8	4.0	0.3	3.0	4.2
**Sao Tome e Principe**	2008	1.6	20.7	26.9	0.7	3.9	6.8	0.7	4.4	7.4
**Senegal**	2014	0.8	18.5	21.6	0.2	0.5	1.1	0.1	0.2	0.6
**Serbia**	2010	0.6	2.1	4.5	0.9	11.0	14.4	0.5	2.3	4.1
**Sierra Leone**	2013	1.0	31.5	35.5	0.4	2.7	4.2	0.4	3.6	5.2
**Somalia**	2006	1.4	36.9	42.5	0.3	1.5	2.9	0.3	1.8	2.9
**Suriname**	2010	0.7	11.8	14.6	0.6	7.6	10.1	0.3	1.5	2.6
**Swaziland**	2010	1.1	25.8	30.2	0.6	6.8	9.1	0.4	2.2	3.6
**Syria**	2006	0.5	15.9	17.9	0.3	6.5	7.8	0.5	10.0	11.8
**Tajikistan**	2012	0.9	21.3	24.7	0.4	2.0	3.5	0.4	2.4	4.1
**Tanzania**	2009-10	0.9	37.2	40.8	0.3	2.1	3.1	0.2	2.2	3.1
**Thailand**	2005-06	0.7	12.4	15.2	0.3	5.5	6.8	0.2	1.6	2.3
**Timor-Leste**	2009	0.8	52.5	55.6	0.1	0.8	1.4	0.3	3.0	4.2
**Togo**	2010	1.0	27.4	31.5	0.3	0.8	2.0	0.1	0.1	0.5
**Turkey**	2003-04	0.8	12.5	15.5	0.5	6.5	8.5	0.2	0.9	1.6
**Uganda**	2011	1.3	28.9	34.1	0.3	1.4	2.7	0.3	1.2	2.5
**Uzbekistan**	2006	0.6	13.5	16.0	0.5	7.0	9.0	0.4	3.6	5.0
**Vanuatu**	2007	1.6	20.2	26.6	0.4	1.0	2.7	0.5	1.6	3.6
**Zambia**	2013-14	0.7	35.4	37.9	0.2	2.2	2.9	0.2	2.9	3.8
**Zimbabwe**	2010	0.8	27.6	30.6	0.3	2.7	3.9	0.2	1.6	2.5

Supplementary Table 2: Exclusion Criteria of DHS/MICS (as noted in survey reports), JME and AEs.[21,22]

Table 2 summarise exclusion criteria for the JME, AEs as well as the estimates in the Final Survey Reports of DHS and MICS (SREs). The SREs are used to triangulate and provide further insight into the estimate and sample size inconsistencies.

Criteria	SREs	JMEs	AEs
**>= 60 months***	E	E	E
**Missing size at birth**	I	*Not Indicated***	I
**Missing Mother's BMI**	E	*Not Indicated*	I
**Mother not listed in household roster**	E	*Not Indicated*	I
**Biologically implausible z-scores**	E (from affected indicator(s))***	E (from affected indicator(s))	E
**Missing one or more of height, weight or age**	E (from affected indicator(s))	E (from affected indicator(s))	E
**Missing sampling weights**	I^	*Not Indicated*	E

*SRE for Honduras provided for 3-59 years, JMEs use age adjustment method to adjust national estimates in cases were age range is different, but there is no other matching criteria to re-analyse data
***Not Indicated* – no indication in manuals or reports of inclusion or exclusion
***Cases with any flagged cases or missing data are excluded from the affected indicator only
^SREs include cases missing sample weights, but not the number of unweighted cases in the reports

Supplementary Table 3: MICS/DHS Final Survey Report Estimates (SREs) and Sample Size with Percentage Point (PP) and Sample Size Differences between SREs with JMEs and AEs[17,18,19,21,22]:

			Survey Report Estimates (SRE) and Sample Size (n)			Difference in JMEs and Sample Size with Survey Reports			Difference in AEs and Sample Size with Survey Reports		
**Country**	Year	Survey Type	Stunting	Over-weight	n[1]	Stunting Δ PP[2]	Over-weight Δ PP	JME Δ *n*[3]	AE Stunting[4] Δ PP	AE Over-weight[4] Δ PP	AE Δ *n*
**Albania**	2008-09	DHS	19.3	21.7	1289	3.8	1.7	201	-0.1	0	2
**Armenia**	2010	DHS	19.3	15.3	1333	1.5	1.5	68	-0.1	0	-4
**Azerbaijan[5]**	2006	DHS	25.1	12.9	1979	1.7	1	148	0	-0.1	-37
**Bangladesh**	2011	DHS	41.3	1.5	7861	0.1	0.4	209	-0.1	0.1	-214
**Barbados**	2012	MICS	7.7	12.2	368	0	0	35	-0.1	-1	-9
**Belarus[5]**	2005	MICS	2.8	6.7	3018	1.7	3	13	-0.1	0.3	-5
**Belize**	2011	MICS	19.3	7.9	1780 (H/A)<br>1768 (W/H)	0	0	29<br>41	0.1	-0.1	-5<br>7
**Benin[5]**	2006	DHS	43.1	9	13099	1.6	2.4	1529	-0.2	0.2	-853
**Bhutan**	2010	MICS	33.5	7.6	5863	0.1	0	275	0.1	-0.2	-189
**BiH**	2011-12	MICS	8.9	17.4	2137 (H/A)<br>2078 (W/H)	0	0	64<br>103	0	-0.6	50<br>-9
**Bolivia**	2008	DHS	27.1	8.5	8422	0.1	0.2	105	0	0	-706
**Burkina Faso**	2010	DHS	34.6	2.4	6994	0.5	0.4	222	-0.1	0	-462
**Burundi**	2010	DHS	57.7	2.7	3590	-0.2	0.2	54	0.2	0	-140
**Cambodia**	2010	DHS	39.9	1.6	3975	1	0.3	102	-0.8	0.1	-276
**Cameroon**	2011	DHS	32.5	6.2	5860	0.1	0.3	246	-0.5	0	-827
**Central African Republic[5]**	2006	MICS	37.5	4.7	5873	7.6	3.8	2714	0.5	1.3	1317
**Chad[5]**	2004	DHS	40.9	NR[6]	4635	3.9	-	253	3.7	-	-251
**Colombia**	2010	DHS	13.2	4.8	15702	-0.5	0	119	-0.1	0	267
**Comoros**	2012	DHS	30.1	9.3	2762	2.0	1.6	357	-0.2	-0.5	-375
**Congo (Brazzaville)**	2011-12	DHS	24.4	3.3	4951	0.6	0.3	-304	-1.3	0	-476
**Congo Democratic Republic**	2013-14	DHS	42.7	4.1	9030	-0.1	0.3	365	-0.4	-0.1	-971
**Djibouti[5]**	2006	MICS	32.6	10.2	1761	0	3.2	411	-2.3	0.2	-54
**Dominican Republic**	2013	DHS	6.9	7.3	3619	0.2	0.3	72	-0.1	0.2	-432
**Egypt**	2014	DHS	21.4	14.9	13601	0.9	0.8	1303	0.1	0	81
**Ethiopia**	2011	DHS	44.4	1.7	10282	-0.2	0.1	892	-0.1	0.1	-671
**Gabon**	2012	DHS	16.5	7.4	3856	1	0.3	175	-0.5	0.1	-520
**Gambia[5]**	2005	MICS	22.4	2.3	6386	5.2	0.4	38	0	0	-31
**Georgia[5]**	2005	MICS	10.4	15.2	1812	4.3	5.8	112	1.4	1.3	2

**Ghana**	2008	DHS	28	5.3	2525	0.6	0.6	141	-0.5	-0.1	-146
**Guinea**	2012	DHS	31.2	3.6	3531	0.1	0.2	151	-0.3	0	-446
**Guinea-Bissau**[5]	2006	MICS	40.9	8.5	4325	6.8	8.5	965	1	2.8	47
**Guyana**	2009	DHS	18.2	6.2	1522	1.3	0.5	129	0.5	0.5	38
**Haiti**	2012	DHS	21.9	3.6	4529	0	0	52	-1	0.1	-545
**Honduras**	2011-12	DHS	22.6	5.1	10167[7]	0.1	0.1	52	-0.3	0.2	-194
**India**[5]	2005	DHS	48	1.5	46655	-0.1	0.4	2578	0	0	-5349
**Iraq**[5]	2006	MICS	21.4	9.4	15316	6.1	5.6	993	1.4	1.9	170
**Ivory Coast**	2011	DHS	29.8	3	3581	-0.2	0.2	163	0	-0.1	-381
**Jordan**	2012	DHS	7.7	4.4	5851	0.1	0.3	52	-0.1	0	416
**Kazakhstan**	2010-11	MICS	13	0.6	4987 (H/A)<br>4955 (W/H)	0.1	12.7	28<br>60	0.2	12.6	3<br>-25
**Kenya**	2008-09	DHS	35.3	4.7	5470	-0.1	0.3	256	-0.1	0	-374
**Kyrgyzstan**	2012	DHS	17.7	8.5	4337	0.1	0.5	52	0.1	0.1	-321
**Lao PDR**	2011-12	MICS	44.2	2	10671	-0.4	0	155	-0.3	0	-122
**Lesotho**	2009-10	DHS	39.2	7.2	2086	-0.2	0.1	53	-1.6	0.4	-774
**Liberia**	2013	DHS	31.6	2.9	3520	0.5	0.3	100	-1.4	0	-349
**Madagascar**[5]	2003-04	DHS	50.1	NR	5412	2.7	-	493	-1.2	-	-978
**Malawi**	2010	DHS	47.1	8.3	4849	0.7	0.9	266	0	-0.1	-263
**Maldives**	2009	DHS	18.9	5.9	2513	1.4	0.6	102	-0.9	-0.1	-160
**Mali**[5]	2006	DHS	37.7	NR	11877	0.8	-	893	0.2	-	-1099
**Mauritania**	2011	MICS	29.7	3.2	8668	0	0	-144	-1.3	0	-492
**Mongolia**	2010	MICS	15.3	10.9	3672	0.3	-6.2	-2967	-0.2	-0.2	-13
**Montenegro**[8]	2013	MICS	9.4	22.3	1361 (H/A)<br>1300 (W/H)	0	0	31<br>92	0.3	-1.1	-47<br>13
**Morocco**[5]	2003-04	DHS	22.4	NR	5311	0.7	-	310	0.1	-	66
**Mozambique**	2011	DHS	42.6	7.4	10313	0.5	0.5	478	0.2	0	-1000
**Namibia**	2013	DHS	23.8	3.4	2287	-0.7	0.7	343	-1.8	0.1	-729
**Nepal**	2011	DHS	40.5	1.4	2475	0	0.1	40	-0.2	0	-140
**Niger**	2012	DHS	43.9	3.5	5481	-0.9	-0.5	354	-0.7	-1.1	-710
**Nigeria**	2013	DHS	36.8	4	26190	-0.4	0.9	2602	-0.1	-0.1	-1685
**Pakistan**	2012-13	DHS	44.8	3.2	3466	0.2	1.6	349	-0.4	0.1	-395
**Peru**	2012	DHS	18.1	NR	9168	0.3	-	20	-0.3	-	45
**Rwanda**	2010	DHS	44.2	6.7	4356	0.1	0.4	63	-0.2	0.2	-281
**Sao Tome e Principe**	2008	DHS	29.3	10.5	1544	2.3	1.1	191	0.1	0.4	-90
**Senegal**	2014	DHS	18.7	1.2	5934	0.7	0.1	106	1.6	-0.2	157
**Serbia**[8]	2010	MICS	6.6	15.6	2745 (H/A)<br>2699 (W/H)	0	0	59<br>305	0	-0.3	-87<br>-41
**Sierra Leone**	2013	DHS	37.9	7.5	5094	0	1.4	677	-0.1	0.2	-1025

**Somalia**[5]	2006	MICS	37.8	3.2	5424	4.3	1.5	419	0	0.3	-65
**Suriname**	2010	MICS	8.8	4	2726	0	0	153	6.3	6.7	933
**Swaziland**	2010	MICS	30.9	10.7	2562 (H/A)<br>2560 (W/H)	0.1	0	14<br>16	0	0	-13<br>-11
**Syria**[5]	2006	MICS	22.4	12.4	9578	6.2	6.3	1009	2.1	2.5	180
**Tajikistan**	2012	DHS	26.2	5.9	5080	0.6	0.7	261	-0.1	-0.1	-557
**Tanzania**	2009-10	DHS	42	5	7491	0.5	0.5	161	-0.4	0.2	-699
**Thailand**	2005-06	MICS	11.9	6.9	9409[9]	3.8	1.1	353	0.1	0.1	426
**Timor-Leste**	2009	DHS	58.1	4.7	8171	-0.4	1.1	666	-0.5	-0.1	-627
**Togo**	2010	MICS	29.7	1.6	4668	0.1	0	11	0	0	-43
**Turkey**[5]	2003-04	DHS	12.2	NR	3668	3.4	-	109	2.9	-	358
**Uganda**	2011	DHS	33.4	3.4	2350	0.3	0.4	98	-0.2	0.3	-280
**Uzbekistan**[5]	2006	MICS	14.6	7.3	4691	5	5.5	192	1	1.2	-8
**Vanuatu**	2007	MICS	20.1	2.3	1281	5.8	2.4	77	0.4	0.6	-81
**Zambia**	2013-14	DHS	40.1	5.7	12328	-0.1	0.5	429	-0.1	0.2	-921
**Zimbabwe**	2010	DHS	32	5.5	5260	0.3	0.3	178	-0.9	-0.2	-961

[1]Where different sample sizes were noted for either height-for-age (stunting) or weight-for-height (overweight) they indicator specific sample size is indicated by (H/A) or (W/H) respectively
[2](Δ PP) differences in percentage points between SREs and JMEs/AEs respectively. Positive values indicate the JME/AE rates for stunting and overweight are higher than the SREs and negative values vice versa.
[3] (Δ n) differences in reported sample size for generation of prevalence rates between SREs and JMEs/AEs respectively. Positive values indicate the JME/AE sample size for stunting and overweight are higher than the SREs and negative values vice versa.
[4]Estimates include stuntedoverweight children
[5]SREs used NCHS not WHO 2006 Child Growth Standards, this is likely to skew understanding of differences in estimates between SREs and AEs/JMEs.
[6]Overweight estimates not reported survey report, difference in sample sizes could thus not be estimated
[7]Survey reports indices and sample for 3-59 months only
[8]JME reports the sample size for weight-for-age as listed in the MICS Final Reports for Montenegro and Serbia. The sample sizes differ for height-for-age and weight-for-height and suggests an error in the JME.
[9]n for all under-fives in dataset, no indication in survey report of final sample size (after exclusion/cleaning) for these estimates, it only lists the weighted population size of 4632212

Other potential sources of the estimate inconsistencies include: 1) error in use/reporting of data, 2) differential application of sampling weights. There remains an issue of transparency in the use of data, and it is not possible to ascertain the consistency of weighting strategies across the three groups of estimates (SREs, JMEs and AEs). Sampling procedures are described in each survey's final reports, yet how the weights for JME or DHS/MICs estimates were finally applied are not currently made publicly available for either the SREs or JMEs. It should be noted that is not possible to verify whether there was incorrect use or reporting of data in the JME or that the exclusion criteria for the JME was applied as described, as this information is also not publicly available.
